# Are YouTube videos on treatments for temporomandibular disorders reliable?: An observational study

**DOI:** 10.1097/MD.0000000000041213

**Published:** 2025-01-03

**Authors:** Mevlüde Yüce Polat, Günay Yapici Yavuz, Onur Odabaşi

**Affiliations:** a Department of Orthodontics, Faculty of Dentistry, Harran University, Sanliurfa, Turkey; b Adiyaman Oral and Dental Health Center, Ministry of Health, Adiyaman, Turkey; c Department of Oral and Maxillofacial Surgery, Faculty of Dentistry, Ankara Yildirim Beyazit University, Ankara, Turkey.

**Keywords:** social media, temporomandibular disorders, YouTube

## Abstract

Today, people frequently turn to the internet to seek information about temporomandibular joint disorders and treatments, as in all other health areas. However, does the information presented online without professional evaluation truly reflect the facts, and if so, to what extent? Based on this question, our study aims to evaluate YouTube™ videos on the treatment of temporomandibular joint diseases. In this cross-sectional study, a search was conducted on YouTube using the search term “TMJ (temporomandibular joint) treatment” for YouTube videos. One hundred sixty-three videos that met the study criteria were evaluated for content usefulness by 3 researchers. The videos were categorized as having low and high content according to usefulness score. All videos were classified according to the source and type of the videos. Statistical analysis were conducted using with Chi-Square test and Mann–Whitney *U* test. It was found that 130 videos had low content, while 33 videos had high content. It was observed that the number of views, duration in minutes, number of comments, number of likes, number of days since uploading and the rate of views were higher in videos with high content (*P* < .05). However, no significant association was found between the usefulness score and the source that uploaded the video and video type (*P* > .05). The results of our study reveal that the vast majority of videos on the treatment of temporomandibular diseases on YouTube contain insufficient information.

## 1. Introduction

Temporomandibular joint disorders (TMD) describe a group of musculoskeletal and neuromuscular disorders affecting the bony components, masticatory muscles, and related tissues of the temporomandibular joint.^[1]^ In a recent meta-analyses published by Valesan et al in which the target point was to evaluate the prevalence of TMD among the general population it was stated that the overall prevalence of TMD was approximately 31% for adult and 11% for adolescents.^[2]^

Today, the internet has made accessing information easier than ever before. An increasing number of users worldwide are using the internet to access health information.^[3]^ As a matter of fact, seeking health information is now among the most popular online activities and the internet is the most popular source for health information.^[4–6]^

When used by healthcare professionals, the internet offers an unprecedented opportunity to share accurate information. However, the internet is a public domain and it is often not possible to check the accuracy of the information shared. As a result, a substantial amount of misinformation is shared and spreads easily. This poses a significant risk to human health and psychology.^[7,8]^ Given the prevalence of temporomandibular diseases and their impact on patients’ daily lives, it is likely that those affected will turn to the internet for information. To the best of our knowledge, only 1 publication^[9]^ in the literature addresses TMD with video analysis on YouTube. However, to our knowledge, no study has specifically investigated TMD treatments on YouTube.

One of the most popular platforms for patients seeking information is YouTube, a free video-sharing site. YouTube has 2.6 billion monthly active users in 2022, and an average of 500 hours of video is uploaded to this platform every minute.^[10]^ Health-related videos uploaded to YouTube are not peer-reviewed and therefore the reliability and scientific accuracy of these videos are seriously questionable.

The aim of our study is to evaluate the quality of YouTube™ videos related to TMD’s treatment and to analyze this quality difference according to the video characteristics. We hypothesize that YouTube videos with higher viewer engagement (e.g., view count, likes, and comments) will have higher content quality and reliability compared to videos with lower engagement.

## 2. Methods

To achieve our research objective, we designed and conducted a cross-sectional study. We used the “Google Trends” search engine to find which keyword was more effective to find the videos published on TMD treatment. Google Trends analysis revealed that “TMJ Treatment” is the most frequently used search term on this topic. We then ran a search for “TMJ treatment” using “sort by relevance” as the default filter for YouTube searches in May 21, 2024. To avoid potential bias from personalized algorithms, we accessed YouTube in incognito mode in the web browser.

It has been reported that most users, when conducting an internet search, review the first 60 to 200 videos, although most YouTube users only watch the first 30 videos.^[11]^ Based on this information, in our study, the first 200 videos were evaluated according to their titles, and it was determined that 191 videos fit the scope of the study. The inclusion criteria are: (1) videos in English; (2) high-quality videos; (3) videos related to the subject. The exclusion criteria are: (1) videos not in English language; (2) low quality videos; (3) irrelevant videos.

Number of views, duration in minutes, number of comments, number of likes and dislikes, interaction index, days since upload and viewing rate were recorded for each video. Again, for each video, the country where the video was uploaded, the source of the video (healthcare professionals, healthcare company, layperson, and other) and the treatment method mentioned in the video were recorded. In addition, videos were classified according to their type (educational, patient’s experience, and misinformation).

YouTube videos were evaluated according to the following contents; (1) TMD definition; (2) etiology; (3) clinical symptoms; (4) radiological findings; (5) classification; (6) anatomy; (7) treatment methods; (8) details of methods; (9) indications; (10) contraindications; (11) complications; (12) advantages; (13) prognosis and survival; (14) cost. The presence of each item was scored with 1 point and the absence with 0 points. Accordingly, a total of 0 to 7 points was determined as low content and was defined as a useless video. It was determined as high content between 8 to 14 points and defined as useful video.

Data were analyzed with IBM SPSS V23. Normality was assessed using the Kolmogorov–Smirnov test. Mann–Whitney *U* test was used to compare data that did not show normal distribution. The data that did not show normal distribution were given as median (minimum–maximum). Fisher Exact test was used for comparison of categorical data. Significance level was taken as *P* < .05. The study was exempted from ethics committee approval as it utilized only publicly available open-source internet data.

## 3. Results

**Figure 1.**
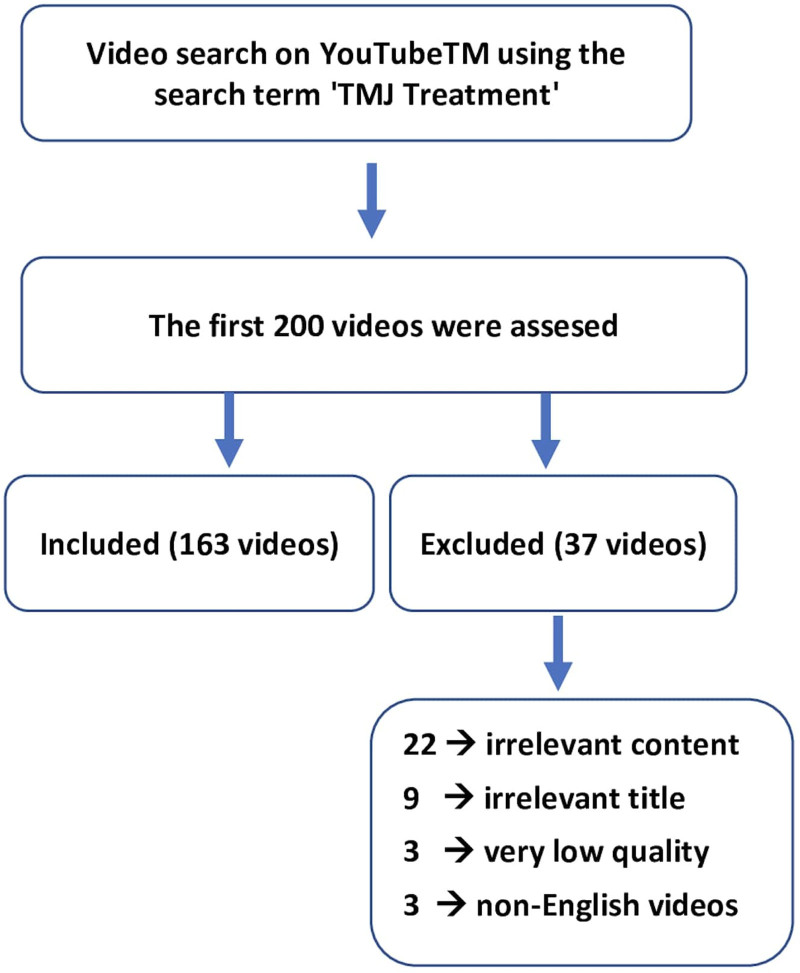
YouTube video selection for analysis.

The first 200 videos were screened based on their titles, and 9 videos were excluded for being irrelevant to the topic. The remaining 191 videos were watched, and 22 were excluded due to irrelevant content, 3 for having low sound and video quality, and 3 for being in languages other than English. The remaining 163 videos were included in the study (Fig. 1).

Based on the usefulness score, 130 videos were categorized as low content (0–7 points) and 33 videos as high content (8–14 points), with a notably high number of low-content videos.

The average number of views of 163 videos is 105.224, the average video duration is 7.007 minutes, the average number of comments is 120.153, the average number of like 1172.503, the average of number of dislike 40.28, the average of interaction index is 0.985, the average of viewing rate is 10065.48. All descriptive statics are shown in Table 1.

**Table 1:** Descriptive statistics.

	Mean ± SD	Median	Minimum	Maximum
Number of views	105,224 ± 277231.5	7891	62	1,752,589
Duration	7.007 ± 7.896	4.25	0	58.39
Comments	120.153 ± 374.353	13	0	3190
Like	1172.503 ± 374.353	59	0	34,451
Dislike	40.282 ± 108.061	3	0	796
Interaction index	0.985 ± 0.790	0.804	-0.071	4.906
Days since upload	1163.859 ± 825.059	992	66	4041
Viewing rate	10065.48 ± 30868.54	816.856	13.802	290484.1

SD = standard deviation.

The majority of the videos were uploaded by healthcare professionals (64.4%, n = 105), followed by healthcare companies (16.6%, n = 27), laypersons (8.6%, n = 14), and other sources (10.4%, n = 17). 83.44% (n = 136) of the videos were educational, 14.72% (n = 24) were patient’s experience, and 1.84% (n = 3) were misinformation videos. When comparing the distribution of video source (*P* = .491) and video type (*P* = .262) between the video quality groups, no statistically significant difference was found (Table 2).

**Table 2:** Comparison of variables between high and low-content videos.

	Usefulness score		Test statistics	*P*-value
	Low (n = 130)	High (n = 33)		
Source				
Healthcare professionals	80 (61.5%)	25 (75.8%)	2.409	.491
Healthcare companies	23 (17.7%)	4 (12.1%)		
Layperson	13 (10%)	1 (3%)		
Other	14 (10.8%)	3 (9.1%)		
Video type				
Educational	105 (80.8%)	31 (93.9%)	2.771	.262
Patient’s experience	22 (16.9%)	2 (6.1%)		
Misinformation	3 (2.3%)	0 (0%)		
Treatment				
Splint	25 (19.2%)	12 (36.4%)	10.255	.215
Medical treatment	8 (6.2%)	6 (18.2%)		
Physiotherapy	64 (49.2%)	18 (54.5%)		
Surgery	6 (4.6%)	6 (18.2%)		
Botulinum toxin injection	5 (3.8%)	3 (9.1%)		
Other	20 (15.4%)	4 (12.1%)		
Dental	26 (20%)	7 (21.2%)		
Athrocentesis	4 (3.1%)	0 (0%)		
Laser	2 (1.5%)	0 (0%)		

In terms of treatment options mentioned in the all videos, the most frequently discussed were physiotherapy at 54.3% (n = 82), splint usage at 24.5% (n = 37), and dental treatments at 21.9% (n = 33). When comparing the treatment options between high and low-quality videos, no statistically significant difference was observed (*P* = .215) (Table 2).

When the groups were evaluated based on YouTube characteristics; in the high score group, it was determined that the number of views (*P* = .012), video duration (*P* < .001), number of comments (*P* = .040), number of likes (*P* = .007), days since upload (*P* = .040), and view rate (*P* = .028), were statistically higher. There was no significant difference between the groups in terms of the number of dislikes(*P* = .081), and interaction index (*P* = .988) (Table 3).

**Table 3:** Comparison of YouTube video characteristic between high- and low-content videos.

	Total score				Test statistics	*P*-value
	Low content		High content			
	Mean ± SD	Median (min–max)	Mean ± SD	Median (min–max)		
Number of views	95922.1 ± 263718.9	6101 (62–1,752,589)	141867.5 ± 327092.9	19,971 (245–1,752,070)	1535	**.012**
Duration in minutes	6.3 ± 8	3.45 (0.35–58.39)	9.6 ± 6.9	8.21 (0–28.1)	1241	**<.001**
Number of comments	117.5 ± 396.9	9.5 (0–3190)	130.6 ± 272.3	34 (0–1488)	1648.5	**.040**
Number of likes	1125.2 ± 3952.1	45 (0–34,451)	1358.8 ± 4306	177 (5–24,659)	1486.5	**.007**
Number of dislikes	37.2 ± 106.1	2 (0–796)	52.3 ± 116.3	8 (0–616)	1728	.081
Interaction index	0.97 ± 0.77	0.81 (-0.07–4.91)	1.04 ± 0.88	0.8 (0–3.67)	2141.5	.988
Days since upload	1070.8 ± 724.7	919.5 (66–3116)	1530.3 ± 1074.7	1530 (95–4041)	1648.5	**.040**
Viewing rate	9978.7 ± 32859.3	566.37 (13.8–290484.09)	10407.4 ± 21704.1	3293.15 (60.33–114514.38)	1614	**.028**

The bold values indicates statistically significant results (*P* < .05).

SD = standard deviation, Min = minimum, Max = maximum.

## 4. Discussion

This study aimed to evaluate the quality of videos on YouTube about the treatments of TMJ diseases. Our study revealed that the source of the video and the video type are not directly related to the content quality of the videos. The fact that a very high percentage (61.5%) of videos with low content quality were uploaded by health professionals, in particular, and that the majority of these videos (80.8%) were in the educational video category, is a finding contrary to expectations. In this situation, viewers who want to reach accurate information should know that the source of the video and the video type are not in a direct causal relationship with the video content.

TMD treatments are quite diverse. There are different options ranging from noninvasive medical treatment to open joint surgery.^[12]^ Only professionals should decide which treatment is appropriate for the patient. However, patients try to guide the physician about treatment options as a result of their false beliefs and sometimes do not accept treatment options that will be beneficial for them. For this reason, it is important to control and analyze health-related information on such video-sharing sites.

The vast majority (n = 130) of the 163 videos included in our study were low-content videos. Besides, only 33 videos had high content. This suggest that a person who searches for information about temporomandibular disorders on YouTube is more likely to encounter low-quality videos. In addition, our study found that 79.75% of TMD-related videos were constructed without including the general definition of the disease, its etiology, clinical and radiological findings, the anatomy of the region, and the classification of the disease.

Our study found that popularity metrics, such as views, likes, and comments, are higher for high-quality videos. A systematic review^[13]^ investigating the reliability of health-related videos on YouTube states that there is no relationship between the number of views and video quality in 23 studies, and that the number of views in 13 studies is higher in low-quality videos (negative correlation), and only in 7 studies that the number of views and likes is higher in high-quality videos (positive correlation). Our study also shows a positive correlation in this sense. This finding suggests that viewers can distinguish accurate, high-quality information on TMJ treatments and show greater interest in it.

When the video uploaders are evaluated in our study, the majority of the uploaders are health professionals (64.4%) and health companies (16.6%). Videos uploaded by individuals are only 8.6% of all videos. In the literature, there are studies stating that layperson uploaded videos are generally based on patient experiences, may contain false information, and are less educational.^[14–16]^ However, the study of Gas et al^[17]^ and Basch et al^[9]^ states that there is no relationship between the source uploading the video and the video quality. In our study, we found that the video source did not affect the video quality, consistent with the findings of Gas et al and Basch et al.^[17]^ Moreover, comparisons of YouTube and peer-reviewed video platforms like WebSurg in other medical fields, such as gynecology, show that peer-reviewed platforms generally offer higher educational reliability and quality for surgical content. These studies highlight the variability in content quality on open-access platforms like YouTube compared to curated, professional sources.^[18–20]^

This study has several limitations. Firstly, YouTube displays videos through an individualized algorithm based on each user’s prior searches. Although we search with the incognito window feature in the internet browser to overcome this limitation, it is still possible to encounter different rankings of videos in searches at different times. Secondly, since YouTube is a very dynamic platform, the number of uploaded videos is constantly renewed, and as a result, the work outputs may differ according to the date of the search. Many videos are added and removed from YouTube every day.

If we go back to the beginning, 1 of the websites that has an important role in spreading false information is YouTube. YouTube generates substantial advertising revenue and compensates content creators based on metrics such as views and likes. In other words, YouTube has become not only a video sharing site, but also a source of income. This means that information sharing is not the only motivation for uploading videos to YouTube. Moreover, YouTube not only contains false information, but also accelerates the spread of this misinformation by encouraging viewers to watch more videos to increase ad revenue.^[13]^

## 5. Conclusion

As a result, YouTube contains a lot of inadequate information as well as providing reliable information on the treatment of temporomandibular diseases. This situation poses a significant risk especially for patients who find it difficult to distinguish between true and false information. For this reason, we think that it is not right for patients to apply to YouTube without the guidance of their physicians. In addition, we suggest that YouTube and similar platforms should develop a mechanism for the content uploaded in the field of health to be published after evaluation by health professionals.

## Author contributions

**Conceptualization:** Mevlüde Yüce Polat, Günay Yapici Yavuz, Onur Odabaşi.

**Data curation:** Mevlüde Yüce Polat, Günay Yapici Yavuz.

**Investigation:** Mevlüde Yüce Polat, Günay Yapici Yavuz, Onur Odabaşi.

**Methodology:** Mevlüde Yüce Polat, Günay Yapici Yavuz.

**Project administration:** Mevlüde Yüce Polat.

**Writing – original draft:** Onur Odabaşi.

**Writing – review & editing:** Mevlüde Yüce Polat, Günay Yapici Yavuz, Onur Odabaşi.
